# Analysis on Two Typical Landslide Hazard Phenomena in The Wenchuan Earthquake by Field Investigations and Shaking Table Tests

**DOI:** 10.3390/ijerph120809181

**Published:** 2015-08-06

**Authors:** Changwei Yang, Jianjing Zhang, Feicheng Liu, Junwei Bi, Zhang Jun

**Affiliations:** 1 School of Civil Engineering, Key of Transportation Tnuuel Engineering, Ministry of Education, Southwest Jiaotong University, Chengdu 610031, China; E-Mails: yangchangwei56@163.com (C.Y.); liufeicheng1@163.com (F.L.); bijunwei1990@163.com (J.B.); 2 Guangxi Key Laboratory of Disaster Prevention and Structural Safety, Guangxi University, Nanning 530004, China; 3 Shanxi Transportation Research Institute, Taiyuan 030006, China; E-Mail: zj_sxjt@hotmail.com

**Keywords:** landslide hazards, slope type, slope angle, shaking table test, field investigations

## Abstract

Based on our field investigations of landslide hazards in the Wenchuan earthquake, some findings can be reported: (1) the multi-aspect terrain facing empty isolated mountains and thin ridges reacted intensely to the earthquake and was seriously damaged; (2) the slope angles of most landslides was larger than 45°. Considering the above disaster phenomena, the reasons are analyzed based on shaking table tests of one-sided, two-sided and four-sided slopes. The analysis results show that: (1) the amplifications of the peak accelerations of four-sided slopes is stronger than that of the two-sided slopes, while that of the one-sided slope is the weakest, which can indirectly explain the phenomena that the damage is most serious; (2) the amplifications of the peak accelerations gradually increase as the slope angles increase, and there are two inflection points which are the point where the slope angle is 45° and where the slope angle is 50°, respectively, which can explain the seismic phenomenon whereby landslide hazards mainly occur on the slopes whose slope angle is bigger than 45°. The amplification along the slope strike direction is basically consistent, and the step is smooth.

## 1. Field Investigations

**Figure 1:**
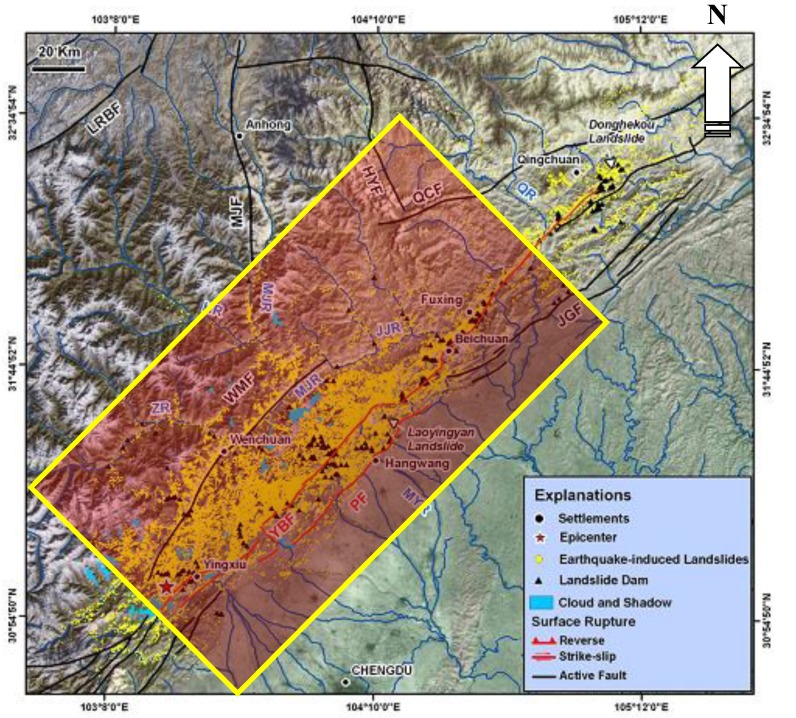
Location and 12 May 2008 Wenchuan earthquake fault surface rupture map.

Earthquake-induced landslides [1,2,3,4,5,6] can result in great damages and losses [7], such as the 2008 Wenchuan earthquake in China [8,9], 1999 Chi-Chi earthquake in Taiwan [10], and 1994 Northridge earthquake in California (USA) [11], *etc.* On 12 of May in 2008, the Ms 7.9 Wenchuan earthquake occurred in the Longmenshan region at the eastern margin of the Tibetan Plateau, adjacent to the Sichuan Basin (See Figure 1), which directly led to 69,200 recorded deaths and 18,195 people are still missing, 21,426,600 homes that badly damaged and more than five million people left homeless [12,13,14]. The earthquake also triggered a large number of landslides, rock avalanches, *etc.*

**Figure 2:**
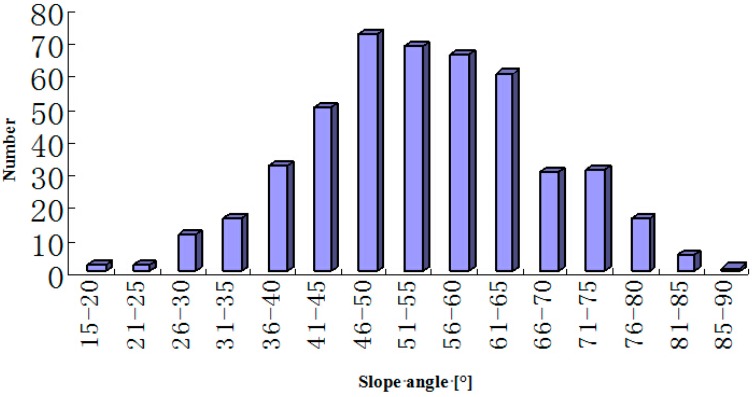
Distribution of landslide points.

**Figure 3:**
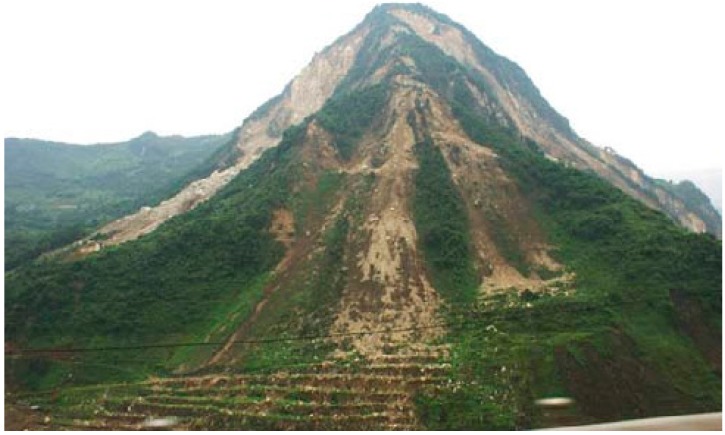
Damage phenomenon of a four sided rock slope in the Wenchuan earthquake.

According to the distribution of the hazards and the landscape conditions, a twenty-three person team headed to disaster zone and a field investigation area was established. The field investigation area (see Figure 1), with a total area of thousands of square kilometers, was carefully investigated. However, because of the traffic, there were some areas that we could not reach, so a total of 1104 large hazards were investigated in detail. Among these hazards, there are 538 collapses, 462 landslides and 104 debris flows, and then a statistical analysis for the landslides according to the slope angle and slope type was made, as shown in Figure 2 and Figure 3. Figure 2 and Figure 3 show that the multi-aspect terrain facing empty isolated mountains and thin ridges was seriously damaged, and the slope angles of most landslides was larger than 45°, which shows that there is some effect of slope type and slope angle on the earthquake responses of slopes, especially acceleration responses, which is the immediate cause triggering landslides.

Aiming at the above disaster phenomena, abundant scientific research has been conducted and abundant findings have been achieved [15,16,17,18,19]. However, this research just focused on the fission laws of single slopes or statistical analyses of the disaster phenomena. Therefore, this article will study the phenomena based on the results from the field investigation and three groups of shaking table tests, in theh hope that the results will help us understand the disaster results.

## 2. Shaking Table Tests

### 2.1. Introduction of the Shaking Table Tests

**Figure 4:**
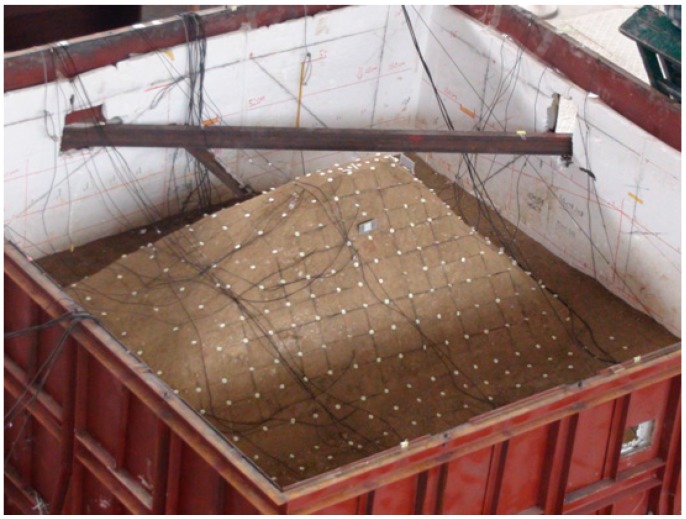
Shaking table test model of an isolated slope.

**Figure 5:**
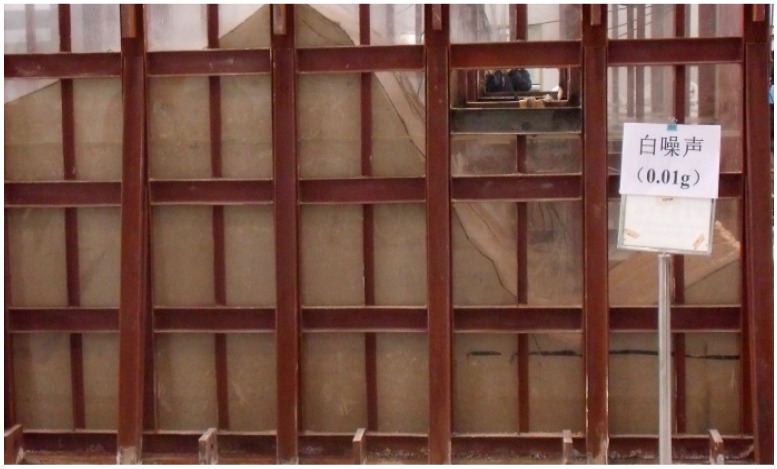
Shaking table test model of one-sided rock slope.

**Figure 6:**
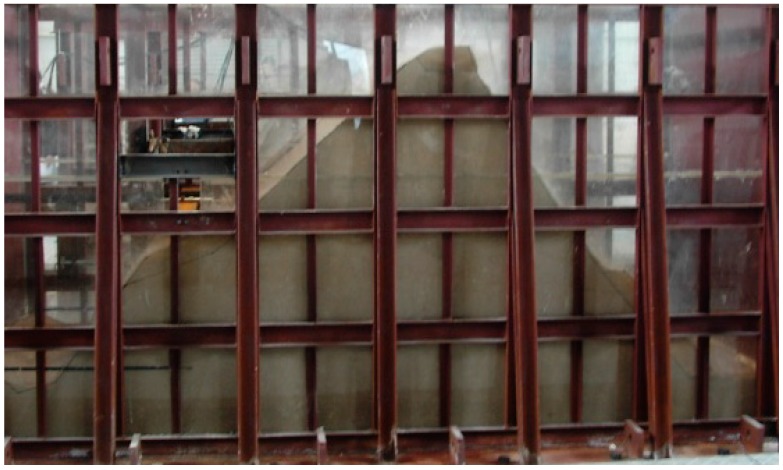
Shaking table test model of two-sided rock slope.

One-sided rock slopes, two-sided rock slopes and four-sided rock slopes are adopted in this shaking table test to clarify the influence of the type of slope surface on acceleration. The test is briefly as follows: in the four-sided slope, the geometric dimension ratio is 1:160, the gravity ratio is 1:1, the time ratio is 1:3.16, the stress ratio is 1:3.16, the Poisson ratio is 1:1 and the internal frictional angle is 1:1. After the calculations based on a similar system, the height of four-sided rock slope is 0.8 m, the length of the bottom is 3.53 m and the width is 1.55 m, while the length of the top surface is 0.78 m and the width is 0.1 m; the four-sides have 30°, 45°, 50° and 60° slopes, respectively, as Figure 4 shows. In the one-sided model and the two-sided model, the geometric dimension ratio is 1:100, the gravity ratio is 1:1, the time ratio is 1:3.16, the stress ratio is 1:6.35, the Poisson ratio is 1:1 and the internal frictional angle is 1:1. After the calculations according to a similar system, the height of the one-sided slope is 1.807 m and the width is 3.5 m, the gradient varies in a range up to 50°, the river valley at the toe of slope is 0.173 m and the river bed inclines slightly, the gradient of the right hand slope is varied in the range from 30°–40°, the top is covered by a seriously weathered layer, as shown in Figure 5. The two-sided slope is 1.8 m high with a width of 3.5 m and includes steep terrain and gentle slope topography; the gradient of the steep terrain is varied in a range up to 50° and the gradient of the gentle slope is varied in the range up to 40°; on the basis of field investigation results, the soil layer and weathered layer on the surface of the slope are generalized to slope wash at the slope top, slope waist and slope foot, as shown in Figure 6. Descriptions of related shaking table tests of one-sided, two-sided and four-sided rock steep slopes can be found in [20,21,22], and are therefore they are not described in detail in this paper.

### 2.2. Selection of Material and Earthquake Wave and Arrangement of Measuring Points

**Figure 7:**
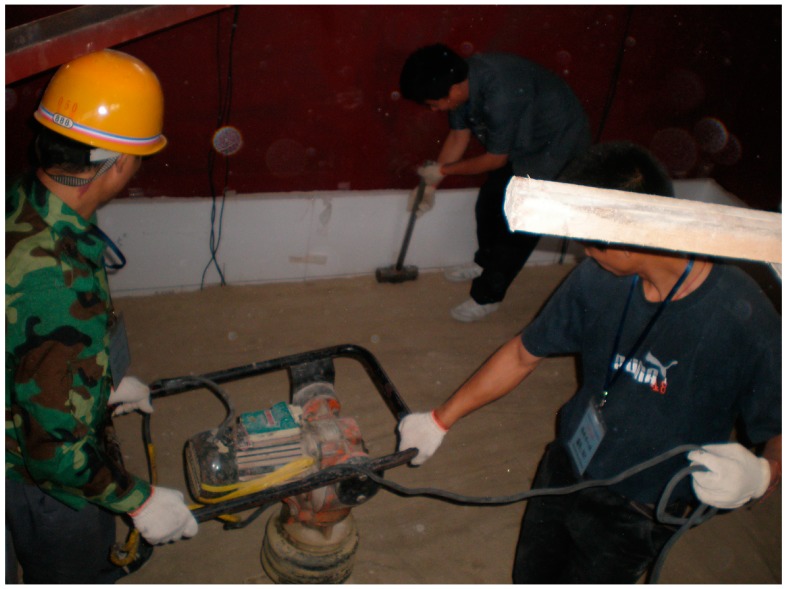
Artificial consolidation and mechanical compaction.

**Figure 8:**
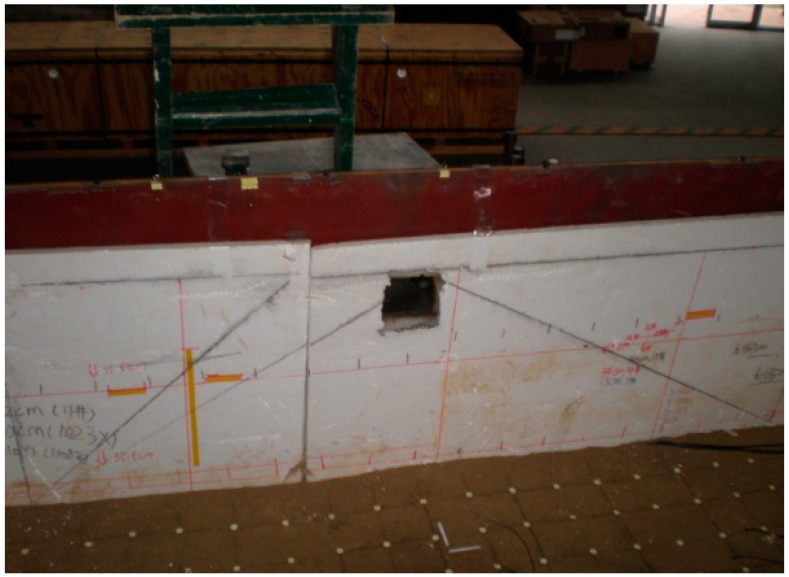
Boundary condition processing.

**Figure 9:**
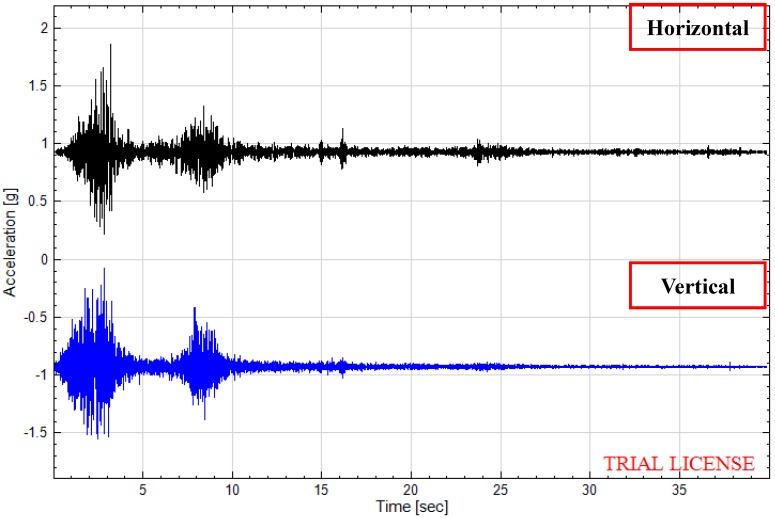
Time history of horizontal and vertical acceleration.

**Figure 10:**
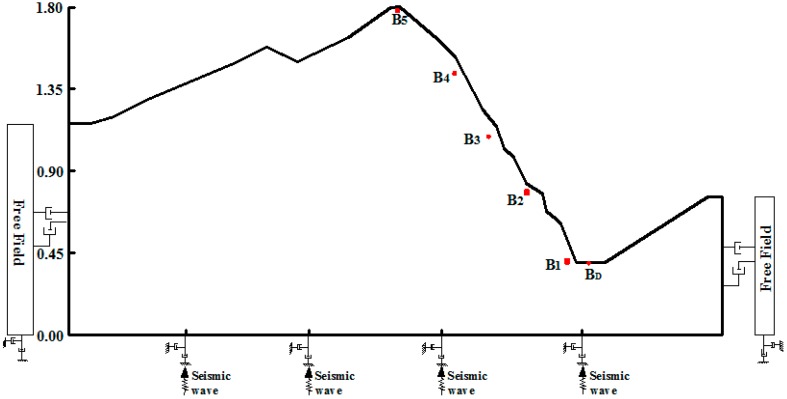
Distribution of monitoring points of the one-sided slope.

**Figure 11:**
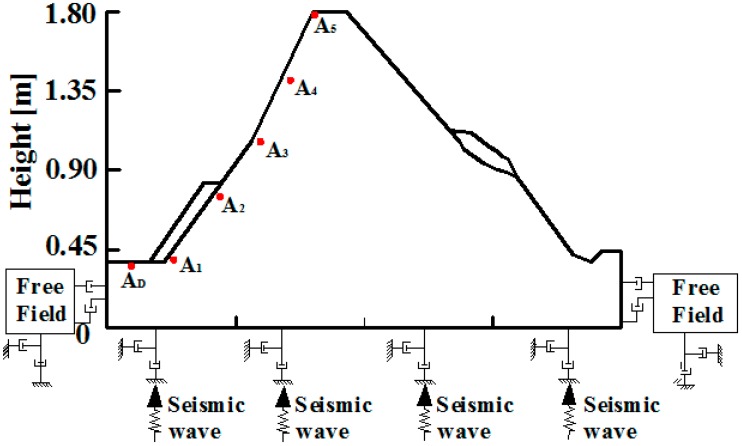
Distribution of monitoring points on the two sided slope.

It is well known that the selection of similar materials plays a vital role in model tests [23]. The materials of the one-sided, two-sided and four-sided slope models which are made up by compounding barite powder, river sand, gypsum, clay and water are chosen by comparison with different similar systems. The ratio of the similar materials in different models and their mechanical parameters are listed in Table 1 and Table 2. With the help of the artificial consolidation and mechanical compaction, the models is set up, as shown in Figure 7. A foam layer with 30 mm width is attached to the inner wall of the model box to eliminate the reflection of waves and as a consequence, eliminate the boundary effect, as Figure 8 illustrates. WenChuan-WoLong earthquake waves, EI Centro earthquake waves and Kobe seismic waves of 0.1 g, 0.2 g, 0.4 g, 0.7 g and 1.0 g can be input into the shaking table, and in this particular case WenChuan WoLong earthquake waves with 0.1 g, 0.2 g, 0.4 g and 0.7 g seismic wave were adopted for this research and corresponding time history curves are displayed in Figure 9. As for acceleration measurement points, five measuring points are arranged in each slope of the one-sided and two-sided rock steep slopes from top to bottom, as shown in Figure 10 and Figure 11, respectively, while four three-dimensional acceleration measuring points are arranged in each slope of the four-sided rock steep slope from top to bottom, as described in [22]. 

**Table 1:** Mechanics parameters of slide mass and slide bed and constitutive model.

Test Model	Model Name	Gravity (kN/m^3^)	Cohesion (kPa)	Internal Friction Angle (°)	Elasticity Modulus (MPa)	Poisson Ratio
One-sided and two-sided rock slopes	Bed rock	22	15.6	37.9	75	0.25
	Slope wash	21	8.3	28.8	10	0.35
Four-sided rock slope	Soft rock	23	20	45	90	0.23

**Table 2:** Ratios of the similar materials in different models (%).

Material	One-Sided Slope		Two-Sided Slope		Four-Sided Slope
	Bedrock	Slope Wash	Bedrock	Slope Wash	Soft Rock
**River sand**	60.40	33.60	60.40	33.60	60.24
**Gypsum**	16.10	35.70	16.10	35.70	16.27
**Clay**	16.10	18.40	16.10	18.40	7.22
**Water**	7.40	12.30	7.40	12.30	16.27

## 3. Effect of Slope Type on Elevation Amplification of Acceleration

The amplification coefficient of acceleration is defined as the ratio of acceleration amplitude in the slope and acceleration amplitude at free field and horizontal acceleration, illustrated as follows: for the one-sided slope model, for example (Figure 10) the x-direction acceleration amplitude of any point A_5_ in the slope is A_X5_, and that of point A_D_ in the free field is A_XD_, then the x-direction acceleration amplification coefficient δ_X_ is written as: δ_X_ = A_X5_/A_XD_. In order to express the influences of the different type slopes on the elevation acceleration amplification effect intuitively, the elevation of each slope is normalized. In addition, the four-sided slope contains four slopes, the gradient of the one-sided slope is nearly equivalent to that of two-sided slope, and nearly equal to 50°, so a slope with 50° gradient is suitable for this analysis, thus slope type is the only influencing factor taken into consideration, ignoring the impact of slope gradient. 

**Figure 12:**
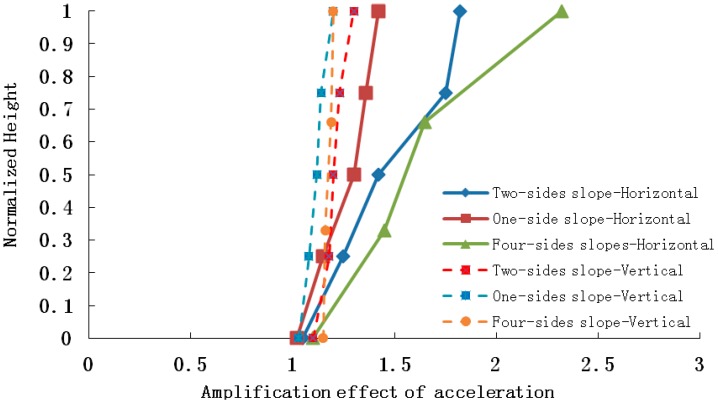
Horizontal and vertical acceleration amplifications when the input is PGA = 0.2 g.

**Figure 13:**
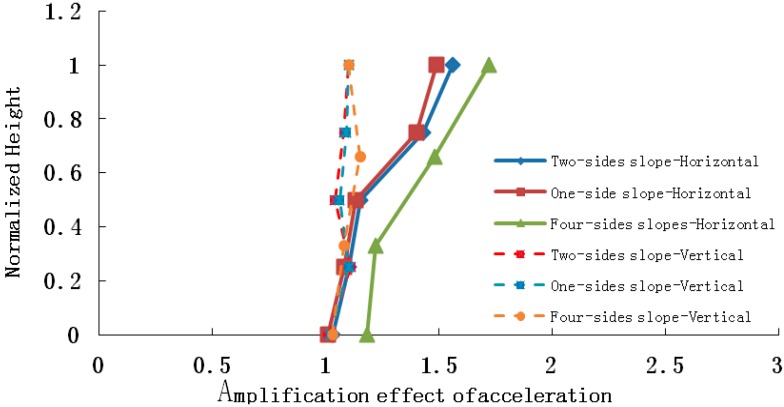
Horizontal and vertical acceleration amplifications when the input is PGA = 0.4 g.

**Figure 14:**
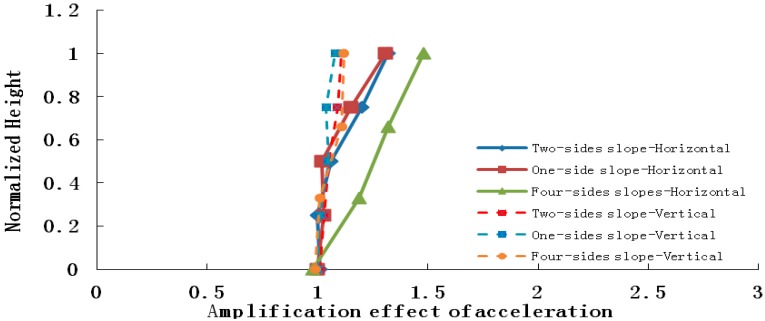
Horizontal and vertical acceleration amplifications when the input is PGA = 0.7 g.

The conclusion drawn from the shaking table test results is that elevation acceleration amplification effect appears not only in the horizontal peak acceleration, but also the vertical peak acceleration, which is shown in Figure 12, Figure 13 and Figure 14.

As the four pictures above show, when 0.2 g, 0.4 g and 0.7 g seismic waves are input into the slope models, the amplification coefficients of the horizontal acceleration of a four-sided rock steep slope varies in the range 1.04–2.15, 1.15–1.89 and 1.00–1.48, respectively, and those of a two-sided rock steep slope are 1.05–1.95, 1.08–1.76 and 1.01–1.32, and those of a one-sided rock steep slope are 1.01–1.60, 1.03–1.62 and 1.00–1.13, while the vertical acceleration amplification coefficients of the four-sided rock steep slope vary in the range 1.05–1.28 ,1.03–1.20 and 1.02–1.12, respectively, and those of the two-sided rock steep slope are 1.05–1.26 , 1.02–1.18 and 1.01–1.11, and those of the one-sided rock steep slope are 1.01–1.15, 1.01–1.16 and 1.01–1.08.Therefore, whatever the slope type, the peak acceleration of the slope increases as elevation increases, and the amplification effect of horizontal acceleration is stronger than that of vertical acceleration, and the amplification effects of a four-sided slope, a two-sided slope and a one-sided slope are ranked in the following order: four-sided slope > two-sided slope > one-sided slope, which explains the phenomenon that two-sided slopes and isolated slopes suffered more serious damage than one-side slopes in the 5.12 Wenchuan earthquake. The primary reasons may be listed as follows: (1) reflected wave splits when the seismic wave propagates to the slope surface, for example: a P wave splits into a P wave and a SV wave after reflection, and a SV wave splits into a SV wave and a P wave after reflection, thus a complex wave field is generated by superposition of reflected waves from different waves, and this causes amplification of the acceleration; (2) the wave field is most evidently split in the four-sided rock steep slope surface, and the weakest in a one-sided slope surface, with the two-sided slope surface laying in between.

## 4. Effect of Slope Angle on Elevation Amplification of Acceleration

**Figure 15:**
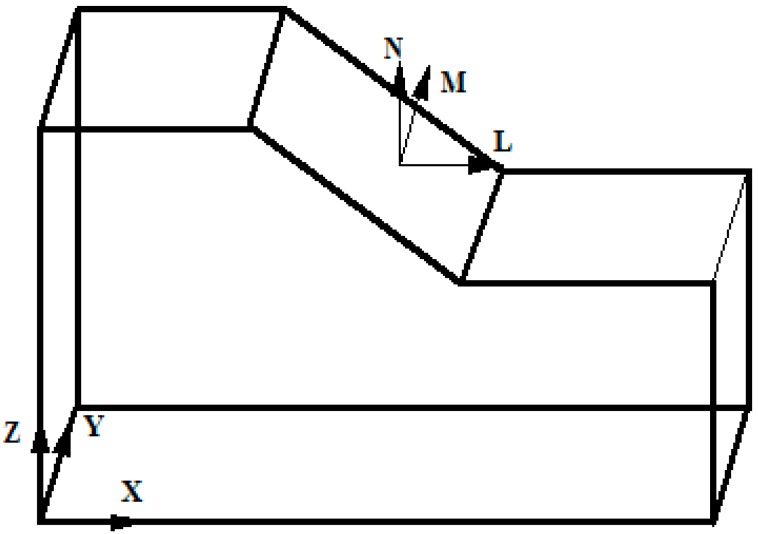
Coordinate system transformation.

**Figure 16:**
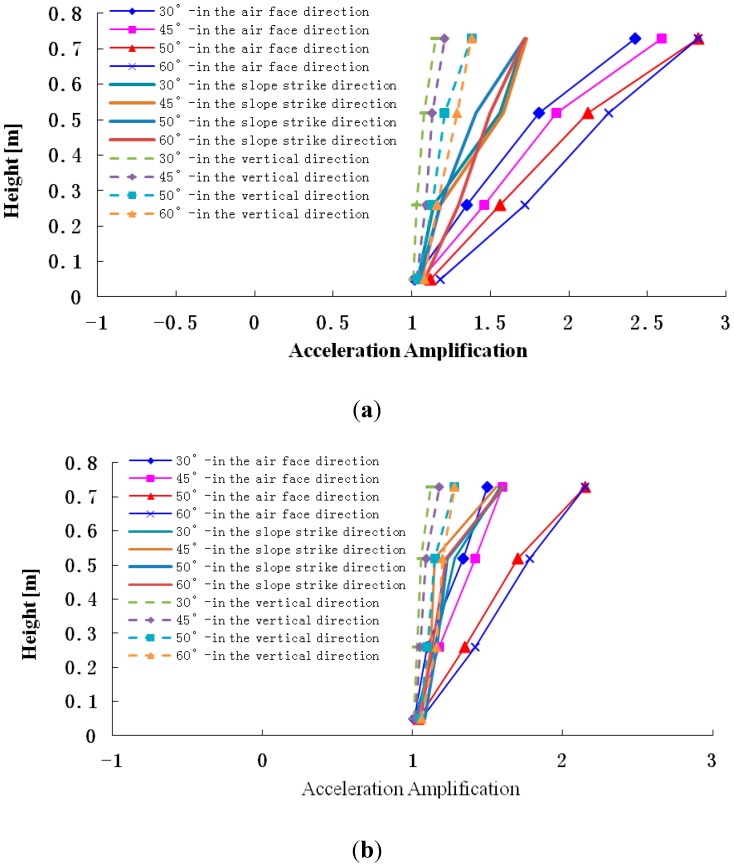

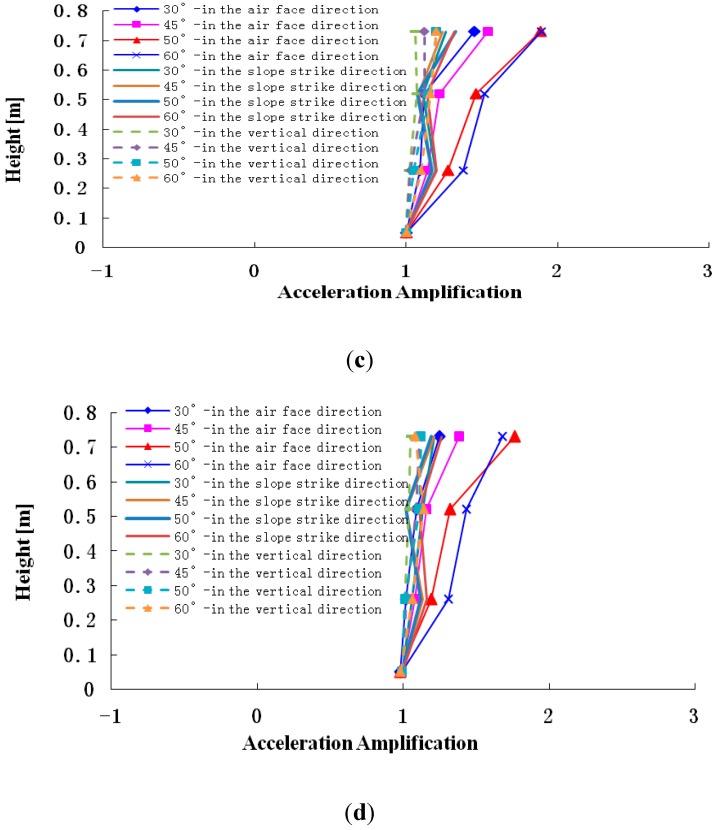
Peak acceleration amplification coefficient in the different directions subjected to different intensity earthquakes. **(a)** Acceleration amplifications in different slope angles and different directions when the input PGA = 0.1 g. **(b)** Acceleration amplifications in different slope angles and different directions when the input PGA = 0.2 g. **(c)** Acceleration amplifications in different slope angles and different directions when the input PGA = 0.4 g; **(d)** Acceleration amplifications in different slope angles and different directions when the input PGA = 0.7 g.

In order to illustrate the effect of slope angle on elevation amplification of acceleration, this paper uses the shaking table test results from the four-sided slope to perform some analysis (Note: if we use X-Y-Z global coordinates for our analysis, which will lead the some problems because this model has four sides, therefore, this paper selects local coordinates that use the surface face direction L, slope strike direction M and vertical direction N to analyze the effect of slope angle on elevation amplification of acceleration, as shown in Figure 15. The results are shown in Figure 16.

Figure 16 shows that when 0.1 g, 0.2 g, 0.4 g and 0.7 g Wenchuan-Wolong seismic waves are input into slope models, the accelerations in the surface face direction L, slope strike direction M and vertical direction N are magnified to different degrees as elevation increases. In the surface face direction L, the acceleration amplification rules can be summarized as follows: 60° slope > 50° slope > 45° slope > 30° slope.

In the slope strike direction M, the acceleration amplification in different slopes is consistent. In the vertical direction N, the acceleration amplification rules can be ranked as follows: 60° slope > 50° slope > 45° slope > 30° slope.

**Figure 17:**
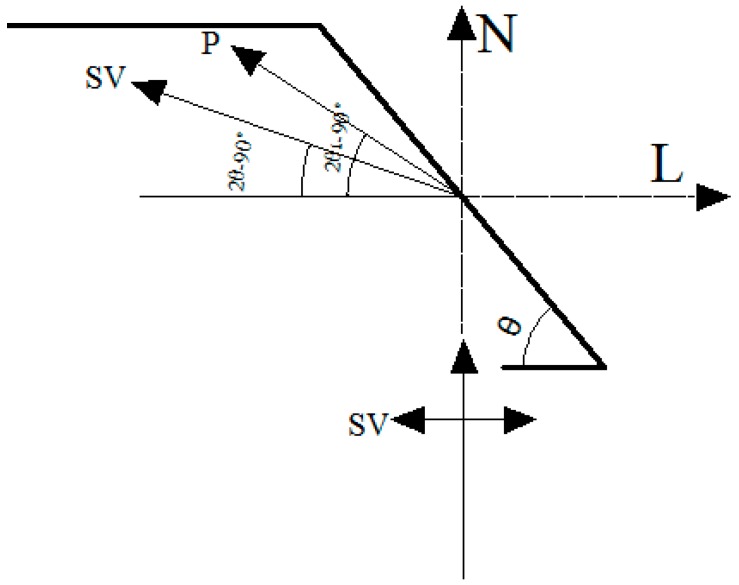
The division of the SV wave field.

Therefore, the acceleration amplification in the surface face direction L and vertical direction N gradually increase as the elevation increases, but that in slope strike direction M is basically unchanged. In order to illustrate the above phenomenon, this paper simplifies the slope problem as a plane strain problem, and illustrates that from the angle of the propagation features of seismic wave, as follows:

According to wave theory, when the SV wave input from the bottom of the model propagates to the slope surface, the reflection waves will produce a wave field separation phenomena, whereby P (SV) waves are transformed into SV (P) waves when the P (SV) wave is reflected on the slope surface, as shown in Figure 17. Therefore, the potential functions of the input SV wave, the reflected SV wave and the reflected P wave are shown as follows:

The input SV wave: The reflected SV wave:

(1) $$\varphi_{i}=E_{s}e^{i(wt-k_{1}x+k_{2s}x_{3})}$$

(2) $$\varphi_{r}=F_{p}e^{i(wt-k_{1}x_{1}-k_{2p}x_{3})}$$

The reflected P wave:

(3) $$\varphi =E_{s}e^{i(wt-k_{1}x_{1}+k_{2S}x_{3})}+F_{s}e^{i(wt-k_{1}x_{1}-k_{2S}x_{3})}$$

where ϕ is the incident angle

(4) $$k_{1}=\frac{\omega \sin\phi }{V_{s}},\;k_{2s}=\{\begin{array}{cc} \sqrt{{\left(\frac{w}{V_{s}}\right)}^{2}-{k^{2}}_{1s}} & k_{1s}\leq \frac{w}{V_{s}}\\ -i\sqrt{{\left(\frac{w}{V_{s}}\right)}^{2}-{k^{2}}_{1s}} & k_{1s}>\frac{w}{V_{s}} \end{array}$$

(5) $$k_{2p}=\{\begin{array}{cc} \sqrt{{\left(\frac{w}{V_{p}}\right)}^{2}-{k^{2}}_{1p}} & k_{1p}\leq \frac{w}{V_{p}}\\ -i\sqrt{{\left(\frac{w}{V_{p}}\right)}^{2}-{k^{2}}_{1p}} & k_{1p}>\frac{w}{V_{p}} \end{array}$$

(6) $$\frac{F_{P}}{E_{S}}=\frac{-2V_{p}^{2}\sin2\phi \cos2\phi }{{V_{s}}^{2}\sin2\theta \sin2\phi +V_{p}^{2}\cos2\phi }$$

(7) $$\frac{F_{s}}{E_{S}}=\frac{V_{s}^{2}\sin2\theta \sin2\phi -V_{p}^{2}\cos2\phi }{{V_{s}}^{2}\sin2\theta \sin2\phi +V_{p}^{2}\cos2\phi }$$

**Figure 18:**
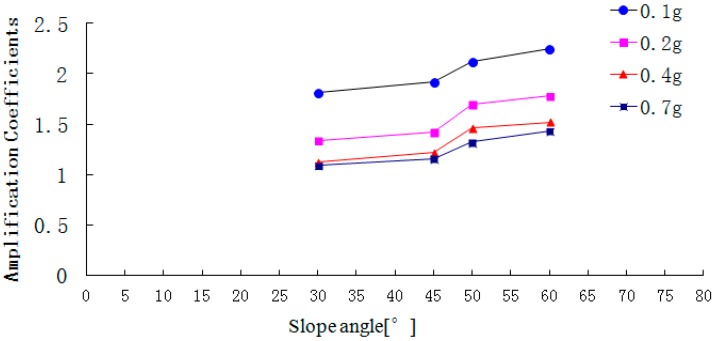
The peak acceleration amplification coefficient on the free face.

**Figure 19:**
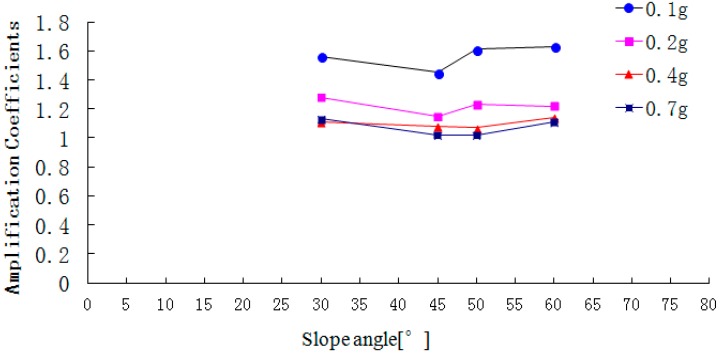
The peak acceleration amplification coefficient in the slope direction.

**Figure 20:**
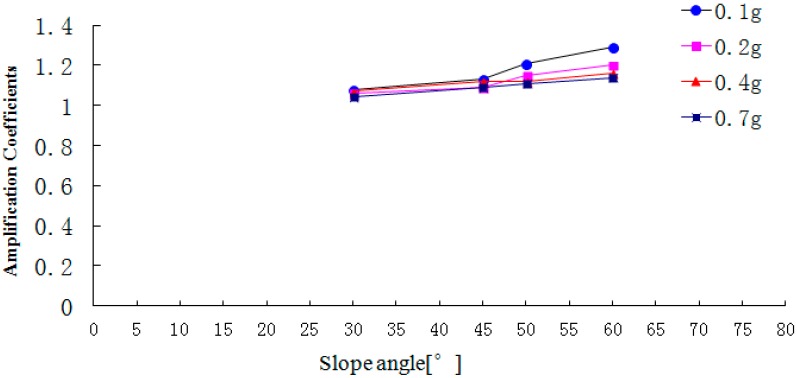
The peak acceleration amplification coefficient in the vertical direction.

Based on the above formulas, we put the related parameters into the formulas, and then the results can be obtained, as shown in the following: F_P_/E_S_ is a negative number, but **F_S_/E_S_** is a positive number. That fully shows that the phase of the input SV wave is consistent with that of the reflected SV wave, but is opposite to that of the reflected P wave. It leads to the fact that the vibration component of the input SV wave and the reflected SV wave in the direction L are consistent with that in the direction N, but that of the input SV wave and reflected P wave in the direction L is opposite to that in the direction N. At the same time, the components of the input SV wave in the direction Land N is E_s_ and 0, respectively. That of reflected SV wave in the direction Land N is (E_S_ + F_S_)sin(2θ − 90°) and (E_S_ + F_S_)cos(2θ − 90°), respectively. That of reflected P wave in the direction Land N is F_p_cos(2θ_1_ − 90°) and F_p_sin(2θ_1_ − 90°), respectively. Therefore, the total vibration components in the direction L and N are E_s_ + (E_S_ + F_S_)sin(2θ − 90°) + F_p_cos(2θ_1_ − 90°) and (E_S_ + F_S_)cos(2θ − 90°) + F_p_sin(2θ_1_ − 90°). In addition, with the increase of incident angle, the reflection angle of the SV wave gradually increases and the vibration direction moves to the direction L. However, the reflection angle θ_1_ of the P wave gradually increases and the vibration component in the direction L gradually decreases. So both (E_S_ + F_S_)sin(2θ − 90°) + F_p_cos(2θ_1_ − 90°) and (E_S_ + F_S_)cos(2θ − 90°) + F_p_sin(2θ_1_ − 90°) gradually increase. Therefore, with the increase of slope angle, the amplification of acceleration in the directions L and N gradually increases. A similar conclusion about the amplification in the direction M can be obtained by using the above thinking. In order to reveal the effect of slope angle on the acceleration amplification clearly, only the measuring point at the position H/4 is selected for analysis, and the results are shown in Figure 18, Figure 19 and Figure 20.

Figure 18, Figure 19 and Figure 20 show that with the increase of slope angle, the acceleration amplification coefficients in the direction L, M and N gradually increase and there is a step in the curve. When the slope angle ranges from 30° to 45° or from 50° to 60°, the acceleration amplification coefficients increase slightly, but from 45° to 50°, the amplification coefficients suddenly increase. In addition, the change law of the step height in the acceleration amplification coefficients curve is ranked as follows: that in the air face direction L> that in the slope strike direction M > that in the vertical direction N. At the same time, when the PGA of the input Wenchuan-Wolong wave is 0.1 g, 0.2 g, 0.4 g and 0.7 g, the acceleration amplification coefficients of the 50° slope in the air face direction are 42.3%, 19.7%, 19.7% and 13.8% larger than that of the 45° slope. Therefore, as the slope angle increases, the acceleration amplification coefficients do not only increase, but have two inflection points. Therefore, the dynamic responses of a rock slope whose slope angle is 45° is stronger than that of rock slope with an angle below 45°, which can explain well the seismic hazard whereby most landslides occur on the slopes whose angle is larger than 45°.

## 5. Conclusions

Considering two kinds of serious disaster phenomena in the Wenchuan earthquake, which are that the multi-aspect slopes facing empty isolated mountains and thin ridges reacted to the earthquake intensely and were seriously damaged and the slope angles of most landslides was larger than 45°, the primary reasons are analyzed on the basis of field investigations and shaking table tests of one-sided, two-sided and four-sided slopes. The following specific conclusions are proposed:

The multi-aspect slopes facing empty isolated mountains and thin ridges react to earthquakes intensely and get seriously damaged and the slope angles of most landslides is larger than 45°. Therefore, we should pay high attention to security protection work on high steep slopes when their slope angle is larger than 45° and multi-aspect slopes facing empty isolated mountains. 

Regardless of whether one-sided, two-sided or four-sided steep slope are considered, the acceleration of different sites is magnified to different degrees as the elevation increases. The horizontal peak acceleration amplification effect is stronger than that of vertical peak acceleration. As for different slope shapes, the amplification effect of acceleration of four-sided slopes is the strongest, and that of one-sided slopes is the weakest, and that of two-sided slopes lays in between, which accounts for the phenomenon that two-sided slopes and isolated slopes were more seriously damaged than one-side slopes in the the 5.12 Wenchuan earthquake. The amplifications of the peak accelerations gradually increase as the slope angles increase, and there are two inflection points which are the point where the slope angle is 45° and where the slope angle is 50°, respectively, which can explain the seismic phenomena whereby landslide hazards mainly occur on the slopes whose slope angle is bigger than 45°. The amplification along the slope strike direction is basically consistent, and the step is smooth.

## References

[1] Yang C.W., Zhang J.J., Fu X., Zhu C.B., Bi J.W. (2014). Improvement of pseudo-static method for slope stability analysis. J. Mt. Sci..

[2] Huang Y., Chen W., Liu J. (2012). Secondary geological hazard analysis in Beichuan after the Wenchuan earthquake and recommendations for reconstruction. Environ. Earth Sci..

[3] Huang Y., Zhang W.J., Xu Q., Xie P., Hao L. (2008). Run-out analysis of flow-like landslides triggered by the Ms 8.0 2008 Wenchuan earthquake using smoothed particle hydrodynamics. Landslides.

[4] Yang C.W., Liu X.M., Zhang J.J., Chen Z.W., Shi C., Gao H.B. (2014). Analysis on mechanism of landslides under ground shaking: a typical landslide in the Wenchuan earthquake. Environ. Earth Sci..

[5] Prestininzi A., Romeo R. (2000). Earthquake-induced ground failures in Italy. Eng. Geol..

[6] Chigira M., Wu X., Inokuchi T., Wang G. (2000). Landslides induced by the 2008 Wenchuan earthquake, Sichuan, China. Geomorphology.

[7] Bird J.F., Bommer J.J. (2004). Earthquake losses due to ground failure. Eng. Geol..

[8] Dai Z.L., Huang Y., Cheng H.L., Xu Q. (2014). 3D Numerical modeling using smoothed particle hydrodynamics of flow-like landslide propagation triggered by the 2008 Wenchuan earthquake. Eng. Geol..

[9] Huang Y., Jiang X.M. (2010). Field-observed phenomena of seismic liquefaction and subsidence during the 2008 Wenchuan earthquake. Natural Hazards.

[10] Khazai B., Sitar N. (2003). Evaluation of factors controlling earthquake-induced landslides caused by Chi-Chi earthquake and comparison with the Northridge and Loma Prieta events. Eng. Geol..

[11] Jibson R.W., Harp E.L., Michael J.A. (2000). A method for producing digital probabilistic seismic landslide hazard maps. Eng. Geol..

[12] Cui P., Zhu Y.Y., Han Y.S., Chen X.Q., Zhuang J.Q. (2009). The 12 May Wenchuan earthquake induced landslide lakes: distribution and preliminary risk evaluation. Landslides.

[13] Zhang J.J., Yang C.W., Zhao J.X., Graeme H.M.V. (2012). Empirical models for predicting lateral spreading with considering the effect of region seismicity. Earthquake Eng. Eng. Vib..

[14] Chen Z.Y., Shen H. (2014). Dynamic centrifuge tests on isolation mechanism of tunnels subjected to seismic shaking. Tunn. Undergr. Space Technol..

[15] Cao Y.B., Dai F.C., Xu C., Tu X.B., Min H., Cui F.P. (2011). Discrete element simulation of deformation and movement mechanism for Tangjiashan landslide. Chin. J. of Rock Mech. Eng..

[16] Li S.H., Liu T.P., Liu X.Y. (2009). Analysis method for landslide stability. Chin. J. of Rock Mech. Eng..

[17] Chen Z.Y., Wei C., Li T.B., Yuan Y. (2012). Damage characteristics and influence factors of mountain tunnels under strong earthquakes. Natural Hazards.

[18] Xiao S.G., Feng W.K., Zhang J.J. (2010). Analysis of the effects of slope geometry on the dynamic response of a near-field mountain from the WenChuan earthquake. J. Mt. Sci..

[19] Yang C.W., Gao H.B., Zhang J.J. (2013). Research on generality and otherness of seismic response of the steep rock slope. J. Sichuan Univ..

[20] Yang C.W., Zhang J.J. (2014). Time-frequency analysis method on elevation amplification effect of acceleration of two-side high steep rock slope. Chin. J. of Rock Mech. Eng..

[21] Han Y.K., Yang C.W., Zhan J.J., Bi J.W., Gao H.B. (2014). The influence of slope angle on elevation amplification effect of rock slope acceleration. J. China Earthquake Eng..

[22] Hong Y.S., Chen R.H., Wu C.S., Chen J.R. (2005). Shaking table tests and stability analysis of steep nailed slopes. Can. Geotech. J..

[23] Lin M.L., Wang K.L. (2006). Seismic slope behavior in a large-scale shaking table model test. Eng. Geol..

